# The road to recovery: impact of COVID-19 on healthcare utilization in South Korea in 2016–2022 using an interrupted time-series analysis

**DOI:** 10.1016/j.lanwpc.2023.100904

**Published:** 2023-09-21

**Authors:** Katelyn Jison Yoo, Yoonkyoung Lee, Seulbi Lee, Rocco Friebel, Soon-ae Shin, Taejin Lee, David Bishai

**Affiliations:** a World Bank Group, South Korea; b Johns Hopkins Bloomberg School of Public Health, USA; c Seoul National University, South Korea; d Hong Kong University, Hong Kong, China; e National Health Insurance Service, South Korea; f London School of Economics and Political Science, England

**Keywords:** National health insurance service, Health service utilization, Interrupted time-series analysis, Impact of COVID-19, Road to recovery, South Korea, Vulnerable populations, Type of healthcare services, Age, Sex, Income level, Health facility type, Avoidable/non-avoidable hospitalizations, Negative binomial model, Low-income level, Aging population, Medicaid, Low-income, Elderly

## Abstract

**Background:**

The COVID-19 pandemic substantially disrupted healthcare utilization patterns, globally. South Korea had been praised widely in its efforts to contain the spread of the pandemic, which may have contributed to a significantly smaller reduction in healthcare utilization compared to neighboring countries. However, it remains unknown how the COVID-19 pandemic impacted utilization patterns across population sub-groups, particularly vulnerable patient groups in South Korea. This paper quantifies the changes in healthcare utilization attributable to COVID-19 and the COVID-19 vaccination by sub-groups.

**Methods:**

An interrupted time series analysis was conducted to examine the impact of COVID-19 on healthcare utilization in South Korea from January 2016 to December 2022 using aggregated patient-level data from the national health insurance system that accounts for 99% of all healthcare services in South Korea. We applied negative binomial models adjusting for seasonality and serial correlation. Falsification tests were conducted to test the validity of breakpoints. Stratified analyses by type of healthcare services, age, sex, income level, health facility type, and avoidable/non-avoidable hospitalizations was performed, and we assessed differences in utilization trends between population groups across three phases of the pandemic.

**Findings:**

In early 2020, the COVID-19 pandemic caused a reduction in monthly volume of outpatient utilization by 15.7% [95% CI 13.3%–18.1%, p < 0.001] and inpatient utilization by 11.6% [10.1%–13.0%, p < 0.001]. Most utilization recovered and rebounded to pre-COVID-19 levels as of December 2022 although variations existed. We observed heterogeneity in the magnitude of relative changes in utilization across types of services, varying from a 42.7% [36.8%–48.0%, p < 0.001] decrease for pediatrics, a 23.4% [20.1%–26.5%%, p < 0.001] reduction in utilization of public health centers, and a 24.2% [21.2%–27.0%, p < 0.001] reduction in avoidable hospitalizations compared to the pre-pandemic period. Contrary to global trends, health utilization among the elderly population (65 and older) in South Korea saw only marginal reductions compared to other age groups. Similarly, Medicaid patients and lower income groups experienced a smaller reduction compared to higher income groups.

**Interpretation:**

The impact of the COVID-19 pandemic on healthcare utilization in South Korea was less pronounced compared to the global average. Utilization of vulnerable populations, including adults over 65 years old and lowest-income groups reduced less than other type of patients.

**Funding:**

No funding.

**Research in context**

**Evidence before this study**

COVID-19 substantially disrupted the normal patterns of healthcare utilization globally, with a median reduction of 37.2% between the pandemic and pre-pandemic period and ranging from −19.8% to −50.5%.

South Korea has been praised widely in its efforts to contain the spread of the pandemic with one of the lowest mortality rates among Organization for Economic Co-operation and Development (OECD) countries even at the height of the Omicron wave. Successful containment strategies possibly contributed to a significantly smaller reduction in healthcare utilization compared to neighboring countries.

Recent analysis showed that the number of personal hospital visits still decreased by 11.9% in 2020 compared to the previous year. It remains unknown how the pandemic impacted utilization patterns across population sub-groups, particularly vulnerable patients in South Korea.

In terms of the sources, we have referenced all literature databases available online. We also crosschecked our storyline and data from the NHIS reports and databases for triangulation. We focus on data collected post MERS (2015) to remove any impact of MERS on healthcare utilization, thus the timeframe is from 2016.

**Added value of this study**

To our knowledge, this is the first study to examine how Korea's healthcare utilization evolved due to COVID-19 to this granular level. The study is comprehensive as it covers the entire population across 7 years, stratified by sub-group categories, and uses a robust statistical method.

The impact of the COVID-19 pandemic on healthcare utilization in South Korea was less pronounced compared to the global average. Utilization of vulnerable populations, including adults over 65 years old and lowest-income groups reduced less than other type of patients.

**Implications of all the available evidence**

It is important for policymakers to understand causes of utilization changes in population groups that were significantly impacted to make decisions on health policies for better and fairer healthcare systems and to adequately prepare and respond to a future pandemic. Our study provides new insight into South Korea's ‘relative’ preservation of healthcare utilization and may help policymakers and researchers in other countries to consider strategies that could curtail about the impact of COVID-19 and that of future pandemics on healthcare utilization.

## Introduction

The Coronavirus disease (COVID-19) pandemic substantially disrupted the normal patterns of healthcare utilization globally, with a median reduction of 37.2% between the pandemic and pre-pandemic period, and ranging from −19.8% to −50.5%.^1^, ^2^, ^3^ The World Health Organization (WHO) (2020) estimated that all countries suffered from disruption of essential health services, leaving populations unable to seek necessary care with adverse consequences for their physical and mental health status.^4^ South Korea has been praised widely in its efforts to contain the spread of the pandemic with one of the lowest mortality rates among Organization for Economic Co-operation and Development (OECD) countries even at the height of the Omicron wave. Successful containment strategies possibly contributed to a significantly smaller reduction in healthcare utilization compared to neighboring countries.^5^^,^^6^ Recent analysis showed that the number of personal hospital visits still decreased by 11.9% in 2020 compared to the previous year.^7^ It remains unknown how the pandemic impacted utilization patterns across population sub-groups, particularly vulnerable patients in South Korea.

Restrictive measures to control the spread of the COVID-19 pandemic caused disruption to access to healthcare services particularly among vulnerable population groups. A systematic review across 20 countries estimated healthcare utilization was reduced by more than one-third^1^ with reductions more severe in lower-income countries due to inadequate health infrastructure and resources. There were changes in health seeking behavior due to reduced income to cover health expenses, and due to fear of contracting the virus in health facilities. Similarly, studies from China and India attributed a decline in medical use due to the strict nationwide lockdowns and the suspension of health facilities’ non-emergency services.^8^, ^9^, ^10^ Reductions in utilization varied among different socio-economic groups. A recent study based on an online survey examined the influence of socio-demographics on healthcare utilization in South Korea and found that women and those with a lower income level, were more likely to forego healthcare utilization than other groups.^11^^,^^12^

Several factors could explain South Korea's smaller reduction of healthcare utilization. More effective management and control of COVID-19 is a leading explanation.^5^^,^^13^ Primary containment strategies such as social distancing, vaccination, testing, quarantine, and isolation were similar across countries with variation in degrees and approaches^14^, ^15^, ^16^, ^17^; however, South Korea's early success in executing these strategies helped control the spread of the pandemic in 2020–2021. This may have led to its smaller reduction of healthcare utilization. Rigorous action to contain the spread of the virus has been attributed partly due to learning derived from of a previous Middle East Respiratory Syndrome (MERS) outbreak in 2015 and establishing subsequent reforms conducive to controlling similar outbreaks.^5^^,^^13^^,^^18^, ^19^, ^20^ The country relied on a pre-existing legal framework reformed after MERS, financing arrangements, strict policies for wearing face masks, social distancing, and a workforce experienced in outbreak management.^5^^,^^13^ Despite the relative achievements, socioeconomic disparities in the access of healthcare services have been observed in South Korea especially during COVID-19 although the extent remains unknown.^21^

This study examines two interrelated research questions: (i) did changes in healthcare utilization attributable to COVID-19 differ by sub-groups, including age, sex, health facility type, income level as proxied by insurance premiums, types of services (departments), and avoidable/non-avoidable services? We hypothesized that heterogeneity in the magnitude of relative changes in utilization existed across different sub-groups. And (ii) was the achievement of high rates of COVID-19 vaccination associated with changes in healthcare utilization across population groups? We hypothesized attaining World Health Organization (WHO)'s recommended 70% vaccination coverage (full doses) increased the level of healthcare utilization. The paper explores the potentially unequal access of health services among different socio-economic status groups through stratified analysis. We also seek to explore other possible reasons for the smaller than average health care utilization reduction besides lower incidence rates of COVID-19 cases in South Korea.

## Methods

### Data sources

#### Medical claims data

Our primary source of data on healthcare utilization comes from monthly aggregates of medical claims data collected by the National Health Insurance Service (NHIS) in South Korea from January 2016 to December 2022 (NHIS-2022-1-788). We focus on data collected post MERS (2015) to remove any impact of MERS on healthcare utilization. We compared demographic and socioeconomic characteristics, including patient age, health facility type, income level based on premium contributions (interchangeably used as ‘income level’ in the paper), types of services (departments), and avoidable hospitalizations^h^ (see list in Supplementary material A). We selected top six departments which cover approximately 80% of the total healthcare utilization in South Korea. Income levels are divided into six groups based on the quintiles of how much insurance premiums are paid based on income levels and Medicaid patient group. Since the compulsory NHIS program achieved universal health coverage in 1989, our data covers 99.7% of the population. All data on the use of medical institutions and pharmacies are collected and managed by the NHIS.

### Statistical analysis

#### Exploratory analyses

We conducted an exploratory analysis of aggregated monthly claims data. Healthcare utilization is defined as the sum of outpatient visits and inpatient utilization per month. Re-visits and re-admissions for the same patient in each month were recorded as separate visits and contributed to the overall number of visits. Outpatient utilization is defined as the sum of outpatient, emergency department, and pharmacy visits. In South Korea, pharmacy visits are counted as a separate outpatient encounter utilized when a patient visits a health facility and fills a prescription. Trends were plotted to visualize changes in health services utilization patterns disaggregated by population subgroups, to understand any significance and outliers.

#### Interrupted time series (ITS) analysis

A set of stratified interrupted time series (ITS) analyses were performed to examine the impact of COVID-19 on healthcare utilization in South Korea from 2016 to 2022. To find the associations between COVID-19 and healthcare utilization, we applied a count data model of utilization events. We applied negative binomial regression models instead of Poisson models accounting for seasonality. This allowed the conditional variance of the outcome variable to be greater than its conditional mean, thus controlling for observed overdispersion in the data.^8^^,^^22^ The model applied the first-order autoregressive structure with heterogenous variances, labeled as AR (1), and Newey–West Heteroskedasticity and Autocorrelation Consistent (HAC) standard errors to control for serial correlation in the residual errors.^23^, ^24^, ^25^

We divided the time series into a pre-COVID-19 phase and then two phases of the COVID-19 period in order to fit a piecewise regression model with three segments. The ITS model included two breakpoints to assess the impact of both the first arrival of the first cases of COVID-19 and the era when vaccination coverage became prevalent in late 2021. The first breakpoint (‘onset of COVID-19’) was set at January 2020, given that the first case reported in South Korea was in January 2020 and when the government raised the Crisis Alert Level from 1 to 4 (highest). The second breakpoint (‘recovery period’) was set as October 2021 when the vaccination rate (fully vaccinated) achieved 70% coverage across the general population.

To assess the robustness of the ITS model, we conducted two approaches using a falsification test drawing from techniques in both time series analysis and the regression discontinuity literature.^26^, ^27^, ^28^ The first falsification test involved conducting a search for “data-driven” structural breaks in the data using a test for an unknown breakpoint.^26^ We conducted the test using the *xtbreak test* command in Stata which tests for multiple breaks at unknown break dates.^27^^,^^28^ For sensitivity purposes, we included a table which shows the respective IRRs by month in the supplementary material C.

Please, refer to supplementary material B for detailed timeline of events during the pandemic. Stratified analyses by sex, age, income-level, department, health facility type, and avoidable hospitalizations were conducted to assess potential differences in the utilization responses across sub-group variables (SGV).

The following seasonally adjusted piecewise regression equation^1^ specifies the model:

[1] $${\text{In}\left(E\left(Y_{it}\right)\right)=\beta }_{i0}+\beta_{i1}time+\sum_{j=1}^{2}\beta_{ij2}{breakpoint}_{j}+\sum_{j=0}^{2}\beta_{ij3}\left(time*{breakpoint}_{j}\right)+\sum_{m=2}^{12}\beta_{im4}month\;+\;\epsilon_{it}$$

Where, $Y_{it}$ denotes the outcome variables of our study (e.g., monthly outpatient visits by age) for sub-group level variables (SGVs) *i* at time *t* . The second term, ${time}_{t}$ is the time (months) elapsed since the start of the study. ${breakpoint}_{j}$ is a dummy variable representing the two breakpoints post-COVID-19, where ${breakpoint}_{1}$ is the onset of COVID-19 set as January 2020 when the first case of COVID-19 was detected in South Korea and ${breakpoint}_{2}$ is the point of time when the country achieved vaccination rate of 70% (fully vaccinated) which is set as October 2021. month is a dummy variable representing month of the year using January as the reference category to control for seasonal effects. There are 12 months indexed by *m*. $\beta_{i0}$ represents the model intercept, $\beta_{i1}$ is the slope of the outcome variable until the onset of the pandemic, $\beta_{ij2}$ represents the change in the level of outcome that occurs in the period immediately following the breakpoints (compared with the counterfactual), and $\beta_{ij3}$ represents the difference between before COVID-19 and post COVID-19 slopes of the outcome.^29^ For instance, $\beta_{i12}$ represents the change of utilization between the pre-COVID-19 period (January 2016–December 2019) and the first break point period (January 2020–September 2021); $\beta_{i22}$ represents the change of utilization between the first break point period and the second break point period (recovery period). Exponentiated regression coefficients (IRR), 95% confidence intervals (CI) and p-values were estimated using STATA SE version 17. The IRR indicates the magnitude of reduced medical utilization compared to the corresponding reference group. We use the Huber-White sandwich estimators (also known as Huber-White or Eicker-White standard errors) because this option allows for unequal variance across all observation points that might be systematically different across sub-groups.

### Ethics statement

This study was exempt from the approval by the Institutional Review Board (IRB) at Seoul National University (IRB No. E2203/003-001).

## Results

The total number of patients in the analysis increased from 51,522,134 in 2016 to 51,948,854 in 2022. Based on the monthly aggregated data, the characteristics of patients who used healthcare services are presented in Table 1. The total number of health facilities in the analysis increased from 55,948 (6% hospitals, 57% clinics and public health centers, and 37% pharmacies) in December 2016 to 65,139 (same %) in December 2022 (16.4% growth) as shown in Table 1. The total number of health service utilization events in Korea including outpatient visits and inpatient discharges was 1.11 billion (99% of the total healthcare utilization constitutes of outpatient services and remainder are inpatient discharges) in 2020, which decreased from 1.31 billion in 2019, and there was a slow recovery in 2021 (1.14 billion) and reached to pre-pandemic levels at the end of 2022 (1.32 billion). Data showed consistent increases in both outpatient and inpatient monthly health service utilization across the pre-pandemic period (2016–2019). We also observed seasonal patterns, with the highest and lowest utilization occurring in December and February on average, respectively.

**Table 1:** Baseline characteristics.

	2016		2017		2018		2019		2020		2021		2022	
	N	%	N	%	N	%	N	%	N	%	N	%	N	%
**Total**	51,522,134	100	51,681,133	100	51,812,088	100	52,032,365	100	51,987,292	100	52,013,073	100	51,948,854	100
**Age group**														
0–6 years	3,082,616	5.98	2,974,088	5.75	2,839,381	5.48	2,658,562	5.11	2,493,483	4.8	2,314,459	4.45	2,130,076	4.1
7–18 years	6,113,346	11.87	5,948,254	11.51	5,805,590	11.21	5,657,805	10.87	5,540,538	10.66	5,486,298	10.55	5,442,023	10.48
19–39 years	15,019,595	29.15	14,898,358	28.83	14,832,012	28.63	14,720,235	28.29	14,404,970	27.71	14,123,232	27.15	13,789,076	26.54
40–64 years	20,276,879	39.36	20,459,405	39.59	20,628,068	39.81	20,905,856	40.18	20,982,328	40.36	21,077,427	40.52	21,086,790	40.59
65–74 years	3,982,715	7.73	4,108,176	7.95	4,259,092	8.22	4,501,866	8.65	4,859,106	9.35	5,158,313	9.92	5,435,129	10.46
75+ years	3,046,983	5.91	3,292,852	6.37	3,447,945	6.65	3,588,041	6.9	3,706,867	7.13	3,853,344	7.41	4,065,760	7.83
**Sex**														
Male	25,797,443	50.07	25,869,547	50.06	25,934,090	50.05	26,051,422	50.07	26,008,094	50.03	25,982,683	49.95	25,927,610	49.91
Female	25,724,691	49.93	25,811,586	49.94	25,877,998	49.95	25,980,943	49.93	25,979,198	49.97	26,030,390	50.05	26,021,244	50.09
**Income Level**														
Medicaid^a^	1,525,727	2.96	1,499,104	2.90	1,530,168	2.95	1,478,123	2.84	1,488,117	2.86	1,520,474	2.92	1,509,035	2.93
0–20th	7,332,696	14.23	7,608,393	14.72	7,926,322	15.30	8,897,447	17.10	8,594,270	16.53	8,249,669	15.86	8,309,092	16.15
21–40th	7,890,027	15.31	7,561,477	14.63	7,407,545	14.30	6,878,493	13.22	7,274,688	13.99	7,746,207	14.89	7,649,763	14.86
40–60th	9,201,597	17.86	9,292,826	17.98	9,216,766	17.79	9,153,815	17.59	9,180,294	17.66	9,179,141	17.65	9,175,443	17.83
60–80th	11,478,650	22.28	11,516,195	22.28	11,491,906	22.18	11,463,642	22.03	11,198,937	21.54	11,166,483	21.47	11,125,250	21.62
80–100th	14,093,437	27.35	14,203,138	27.48	14,239,381	27.48	14,160,845	27.22	14,250,986	27.41	14,151,099	27.21	13,694,058	26.61
**Number of Health facilities**														
Level 3 Hospital^b^	43	0.08	44	0.08	46	0.08	46	0.08	46	0.08	45	0.07	45	0.07
Level 2 Hospital^c^	297	0.53	299	0.53	307	0.53	312	0.53	321	0.53	319	0.53	329	0.51
Level 1 Hospital^d^	1482	2.65	1495	2.63	1437	2.46	1454	2.46	1477	2.46	1354	2.23	1439	2.21
Clinic^e^	28,578	51.08	29,218	51.33	29,913	51.82	30,651	51.82	31,171	51.89	31,583	52.01	33,873	52
Long-term Care Facilities^f^	1398	2.50	1448	2.54	1526	2.63	1554	2.63	1552	2.58	1673	2.75	1755	2.69
Public Health Center^g^	3440	6.15	3436	6.04	3431	5.81	3435	5.81	3301	5.49	3193	5.26	3394	5.21
Pharmacies	20,710	37.02	20,987	36.87	21,332	36.68	21,698	36.68	22,203	36.96	22,560	37.15	24,304	37.31

a Tax-financed medical assistance provided for low-income families.

b Hospitals specializing in high-level medical treatment for severe diseases, referred to tertiary and teaching hospitals in Korea.

c Hospitals equipped with 100 or more beds, 7 or 9 or more medical subjects, and specialists exclusively in each medical subject, referred to general hospitals in Korea.

d Hospitals equipped with 30 or more beds or nursing beds, referred to hospital in Korea.

e Health facilities providing comprehensive medical care with integrated prevention and treatment through patient initial contact.

f Medical institutions where elderly patients are hospitalized to get long-term care services.

g Public health institutions established to improve disease prevention, treatment, and public health.

### Unadjusted changes in healthcare utilization during the COVID-19 pandemic

Table 2, Table 3 shows the unadjusted changes in health care utilization from 2016 to 2022. There were significant decreases in outpatient (Table 2) and inpatient health services utilization (Table 3) after COVID-19 compared to pre-pandemic levels.

**Table 2:** Unadjusted differences between before and after COVID-19 monthly average outpatient healthcare utilization by subgroup.

Category	Before COVID-19 (‘16.01–’19.12) (A)	Before COVID-19 Per capita (C)	During COVID-19 (‘20.01–’22.12) (B)	During COVID-19 Per capita (D)	% Absolute Change ((B-A)/A)	% Per Capita Change ((D-C)/C)
**Total**	107,352,911	2.078	99,209,540	1.908	−7.6%	−8.1%
**Age**						
0–6 years	11,391,899	3.944	6,110,050	2.642	−46.4%	−33.0%
7–18 years	8,313,902	1.414	6,325,360	1.152	−23.9%	−18.5%
19–39 years	16,125,557	1.085	14,788,593	1.048	−8.3%	−3.3%
40–64 years	40,340,102	1.961	38,618,538	1.835	−4.3%	−6.5%
65–74 years	16,594,603	3.939	18,041,700	3.503	8.7%	−11.1%
Over 75 years	14,586,850	4.471	15,325,298	3.955	5.1%	−11.5%
**Sex**						
Male	46,961,230	1.812	43,752,084	1.685	−6.8%	−7.0%
Female	60,391,681	2.336	55,457,456	2.132	−8.2%	−8.7%
**Health facility**						
Level 3 Hospitals	3,651,371		3,967,361		8.7%	
Level 2 Hospitals	6,333,609		6,829,874		7.8%	
Level 1 Hospitals	5,794,552		5,299,612		−8.5%	
Clinics	46,522,371		43,332,723		−6.9%	
Long-term care Facilities	280,423		404,924		44.4%	
Public health center	987,514		463,930		−53.0%	
Pharmacies	43,783,072		38,911,116		−11.1%	
**Income level**						
Medicaid	5,808,805	3.851	5,733,789	3.808	−1.3%	−1.1
0–20th	15,779,567	1.862	15,613,044	1.862	−1.1%	−6.3%
20–40th	13,675,917	1.753	13,245,712	1.753	−3.1%	−4.7%
40–60th	17,659,618	1.773	16,271,943	1.773	−7.9%	−7.5%
60–80th	23,918,871	1.851	20,659,270	1.851	−13.6%	−11.1%
80–100th	30,385,494	1.980	27,780,970	1.980	−8.6%	−7.6%
**Department**						
General	44,845,688		39,406,569		−12.1%	
Internal medicine	20,711,626		19,902,794		−3.9%	
Orthopedic surgery	10,806,420		10,492,345		−2.9%	
Otorhinolaryngology	5,790,106		4,460,414		−23.0%	
Pediatrics	3,412,342		2,267,253		−33.6%	
Neuro psychiatry	1,525,183		2,076,973		36.2%	
Others	20,261,547		20,997,151		3.6%

**Table 3:** Unadjusted differences between before and after COVID-19 monthly average inpatient healthcare utilization by subgroup.

Category	Before COVID-19 (‘16.01–’19.12) (A)	Before COVID-19 per capita (C)	During COVID-19 (‘20.01–’22.12) (B)	During COVID-19 per capita (D)	% Absolute Change ((B-A)/A)	% Per Capita Change ((D-C)/C)
**Total**	1,010,167	0.020	970,067	0.019	−4.0%	−4.6%
**Age**						
0–6 years	112,538	0.039	71,538	0.031	−36.4%	−20.6%
7–18 years	46,525	0.008	37,855	0.007	−18.6%	−12.8%
19–39 years	157,010	0.011	138,921	0.010	−11.5%	−6.7%
40–64 years	375,575	0.018	366,629	0.017	−2.4%	−4.6%
65–74 years	145,460	0.035	168,486	0.033	15.8%	−5.3%
over 75 years	173,060	0.053	186,637	0.048	7.8%	−9.2%
**Sex**						
Male	471,759	0.018	458,518	0.018	−2.8%	−3.0%
Female	538,409	0.021	511,549	0.020	−5.0%	−5.6%
**Health facility**						
Level 3 Hospitals	217,790		238,020		9.3%	
Level 2 Hospitals	338,840		346,130		2.2%	
Level 1 Hospitals	265,581		215,315		−18.9%	
Clinics	137,033		106,988		−21.9%	
Long-term care facilities	50,923		63,614		24.9%	
**Income level**						
Medicaid	80,419	0.053	77,721	0.052	−3.4%	−3.2%
0–20th	148,023	0.019	152,746	0.018	3.2%	−2.3%
20–40th	127,398	0.017	126,334	0.017	−0.8%	−2.4%
40–60th	171,066	0.019	161,248	0.018	−5.7%	−5.3%
60–80th	220,160	0.019	198,788	0.018	−9.7%	−7.1%
80–100th	262,012	0.018	254,098	0.018	−3.0%	−2.0%
**Avoidable hospitalization**						
Non-avoidable	780,730		790,065		1.2%	
Avoidable	229,437		180,002		−27.5%	
**Department**						
Internal medicine	238,175		249,731		0.1%	
Orthopedic surgery	147,255		139,337		−5.7%	
General surgery	93,482		89,513		−3.0%	
Pediatrics	90,991		60,277		−42.3%	
Obstetrics & Gynecology	63,852		51,153		−17.4%	
Neuro Psychiatry	16,429		14,163		−17.1%	
Otorhinolaryngology	23,397		21,068		−11.4%	
Others	338,909		356,850		−0.3%	

Monthly average outpatient utilization before COVID-19 was 107,352,911 (SD = 7,851,536) and 99,209,540 (SD = 11,778,090) between Jan 2020 and Dec 2022. This translates to approximately 2.1 utilization episodes per capita pre-COVID-19 to 1.9 per capita post-COVID-19. Per capita utilization is only available for total utilization and utilization stratified by age and income level.

From 2016 to 2019, we observed steady annual increases ranging from 0.5% to 3% from the previous year; however, there was a 8.1% and 4.6% unadjusted monthly reduction in total outpatient and inpatient utilization per capita post-COVID-19, respectively, compared to pre-pandemic levels (Table 2, Table 3).

Large variations due to COVID-19 on health utilization by age, health facility, income level and departments were observed. The youngest group (0–6-year-olds) health utilization decreased the most after COVID-19 for both outpatients and inpatients episodes per capita by 33% and 20.6%, respectively. Reductions of lesser magnitude were seen for ages 7–18. We also observe the highest utilization per capita for the youngest and eldest group with approximately 4 visits per capita.

We also observed higher reduction in women compared to men. In addition, utilization by type of health facilities varied. Higher tier health facilities such as tertiary hospitals and clinics increased total utilization, while public health centers, which provides primary and essential healthcare services, decreased by 53% compared to pre-pandemic levels. Long-term care facilities experienced a 44.4% increase post-COVID-19.

Table 2 further demonstrates variations among income level by NHIS premium contributions. In this study, NHIS contribution is the proxy of income level. The unadjusted changes show that the higher economic group had higher reductions in outpatient service utilization compared to pre-COVID-19 levels, with the least reduction shown for Medicaid and poorest (0–20 percentile) patient groups.

### Adjusted estimates of changes in healthcare utilization due to the COVID-19 pandemic

**Fig. 1:**
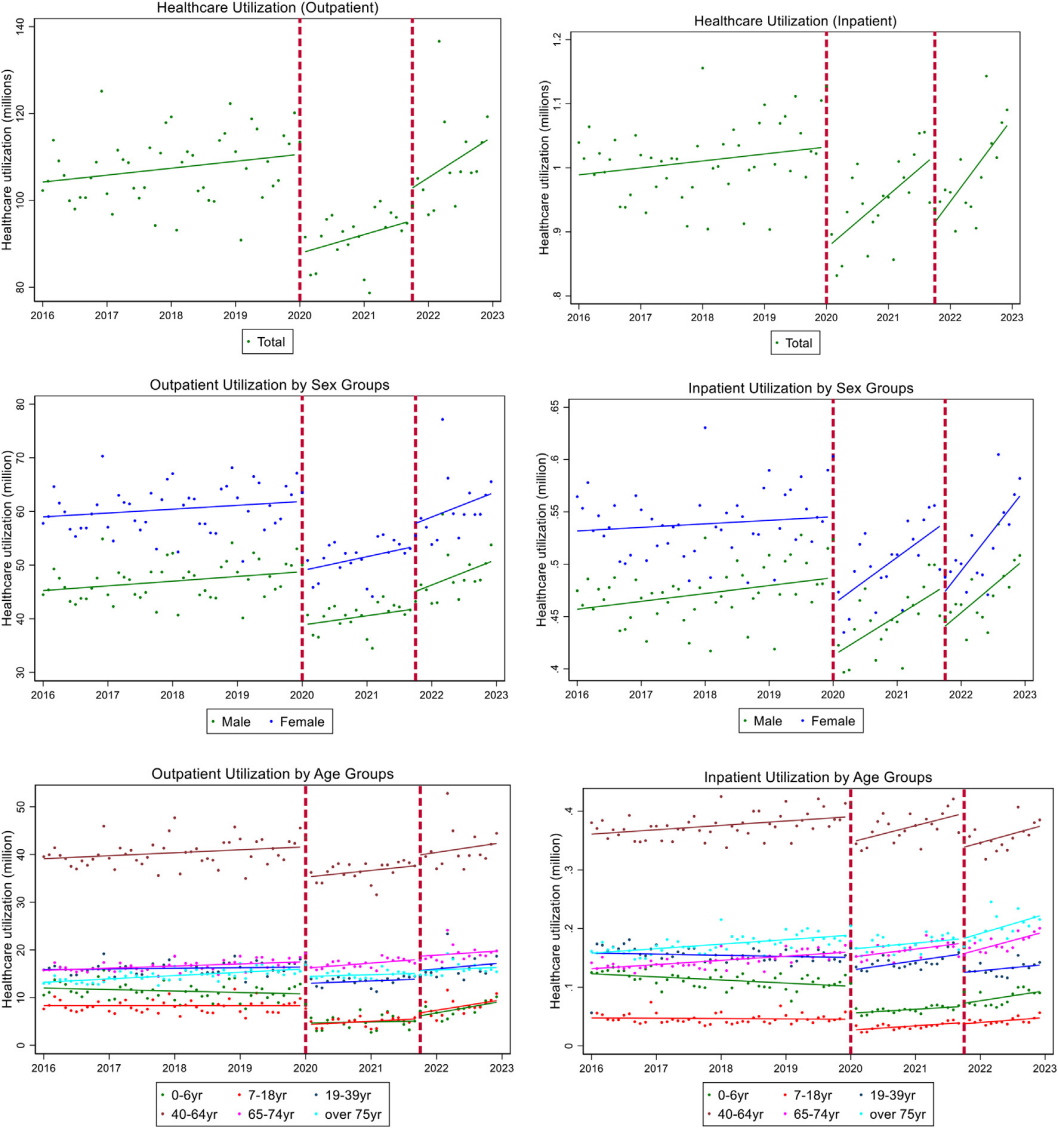

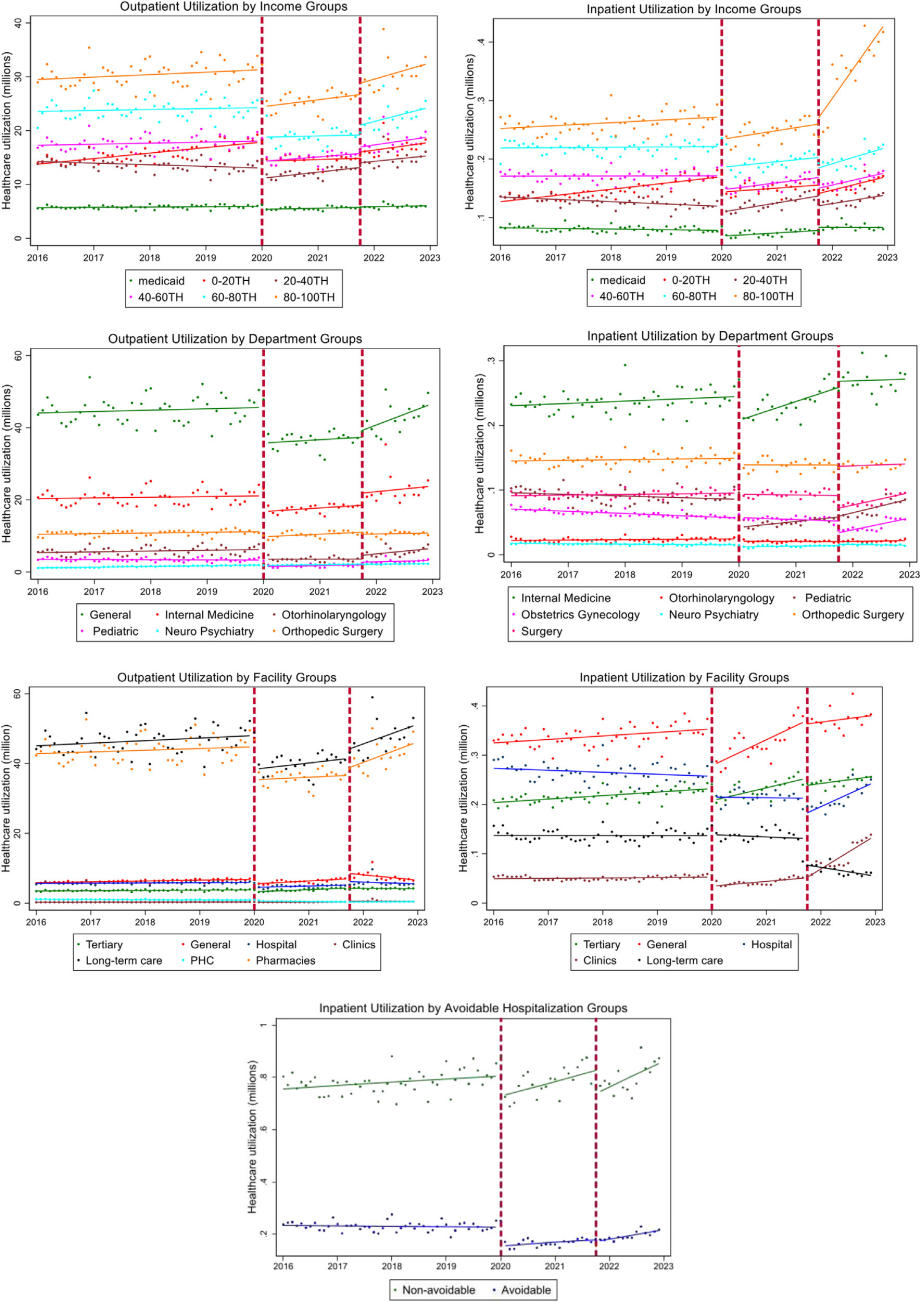
Trends and levels of health care utilization by sub-group levels from 2016 to 2023 in South Korea.

Table 4, Table 5, Table 6, Table 7, Table 8, Table 9, Table 10 shows the adjusted estimates of healthcare utilization changes due to the pandemic examined by different sub-group populations (sex, age, income-level, department, health facility type, and avoidable hospitalizations). The first two IRRs labeled represents $\beta_{2}$ in the model shown in equation^1^ where the first and second IRRs display $\beta_{02}$ and $\beta_{12}$, respectively. Fig. 1 also demonstrates the trends, level changes, and slope by sub-group variables. Respective IRRs for the slope changes are detailed in Supplementary material D.

**Table 4:** Adjusted changes in healthcare services utilization due to COVID-19 and achieving vaccination rate of 70%.

	Onset of COVID-19 January 2020	Recovery Period October 2021 (Achieving vaccination rate 70%)
	IRR (95% CI)	IRR (95% CI)
Outpatient	0.843 (0.819: 0.867)∗∗∗	1.078 (1.048: 1.110)∗∗∗
Inpatient	0.884 (0.870: 0.899)∗∗∗	0.909 (0.893: 0.925)∗∗∗

∗p < 0.1, ∗∗p < 0.05, ∗∗∗p < 0.001.

**Table 5:** Adjusted changes in healthcare services utilization due to COVID-19 and achieving vaccination rate of 70%, stratified by sex.

	Onset of COVID-19 January 2020	Recovery period October 2021 (Achieving second dose vaccination rate 70%)
	IRR (95% CI)	IRR (95% CI)
Outpatient		
Male	0.845 (0.822: 0.870)∗∗∗	1.073 (1.043: 1.104)∗∗∗
Female	0.841 (0.817: 0.865)∗∗∗	1.083 (1.051: 1.116)∗∗∗
Inpatient		
Male	0.887 (0.871: 0.903)∗∗∗	0.941 (0.925: 0.958)∗∗∗
Female	0.882 (0.869: 0.895)∗∗∗	0.882 (0.866: 0.898)∗∗∗

∗p < 0.1, ∗∗p < 0.05, ∗∗∗p < 0.001.

**Table 6:** Adjusted changes in healthcare services utilization due to COVID-19 and achieving vaccination rate of 70%, stratified by age.

	Onset of COVID-19 January 2020	Recovery period October 2021 (Achieving second dose vaccination rate 70%)
	IRR (95% CI)	IRR (95% CI)
Outpatient		
0–6 years	0.548 (0.504: 0.598)∗∗∗	1.239 (1.167: 1.314)∗∗∗
7–18 years	0.642 (0.596: 0.692)∗∗∗	1.250 (1.173: 1.332)∗∗∗
19–39 years	0.850 (0.816: 0.886)∗∗∗	1.139 (1.087: 1.194)∗∗∗
40–64 years	0.887 (0.868: 0.907)∗∗∗	1.061 (1.029: 1.095)∗∗∗
65–74 years	0.943 (0.927: 0.959)∗∗∗	1.032 (1.011: 1.054)∗∗∗
Over 75 years	0.900 (0.888–0.912)∗∗∗	1.033 (1.016: 1.049)∗∗∗
Inpatient		
0–6 years	0.641 (0.587: 0.699)∗∗∗	1.124 (1.057: 1.196)∗∗∗
7–18 years	0.700 (0.648: 0.755)∗∗∗	1.018 (0.958: 1.080)
19–39 years	0.921 (0.892: 0.950)∗∗∗	0.858 (0.814: 0.905)∗∗∗
40–64 years	0.922 (0.914: 0.930)∗∗∗	0.873 (0.857: 0.889)∗∗∗
65–74 years	0.951 (0.936: 0.967)∗∗∗	0.867 (0.854: 0.879)∗∗∗
Over 75 years	0.876 (0.862: 0.890)∗∗∗	0.979 (0.964: 0.995)∗∗∗

∗p < 0.1, ∗∗p < 0.05, ∗∗∗p < 0.001.

**Table 7:** Adjusted changes in healthcare services utilization due to COVID-19 and achieving vaccination rate of 70%, stratified by insurance contribution categories as a proxy for income levels.

	Onset of COVID-19 January 2020	Recovery period October 2021 (Achieving second dose vaccination rate 70%)
	IRR (95% CI)	IRR (95% CI)
Outpatient		
Medicaid	0.923 (0.911: 0.935)∗∗∗	1.021 (1.007: 1.035)∗∗
0–20th	0.843 (0.825: 0.862)∗∗∗	1.076 (1.045: 1.109)∗∗∗
20–40th	0.910 (0.876: 0.944)∗∗∗	1.084 (1.048: 1.121)∗∗∗
40–60th	0.847 (0.821: 0.874)∗∗∗	1.086 (1.053: 1.120)∗∗∗
60–80th	0.819 (0.789: 0.850)∗∗∗	1.082 (1.047: 1.117)∗∗∗
80–100th	0.832 (0.809: 0.856)∗∗∗	1.083 (1.051: 1.116)∗∗∗
Inpatient		
Medicaid	0.884 (0.871: 0.897)∗∗∗	1.099 (1.086: 1.113)∗∗∗
0–20th	0.872 (0.853: 0.891)∗∗∗	0.905 (0.888: 0.922)∗∗∗
20–40th	0.962 (0.939: 0.986)∗∗	0.897 (0.877: 0.918)∗∗∗
40–60th	0.896 (0.880: 0.912)∗∗∗	0.894 (0.875: 0.913)∗∗∗
60–80th	0.866 (0.844: 0.888)∗∗∗	0.919 (0.887: 0.952)∗∗∗
80–100th	0.864 (0.837: 0.891)∗∗∗	0.971 (0.936: 1.006)

∗p < 0.1, ∗∗p < 0.05, ∗∗∗p < 0.001.

**Table 8:** Adjusted changes in healthcare services utilization due to COVID-19 and achieving vaccination rate of 70%, stratified by department (services).

	Onset of COVID-19 January 2020	Recovery period October 2021 (Achieving second dose vaccination rate 70%)
	IRR (95% CI)	IRR (95% CI)
Outpatient		
General	0.843 (0.819: 0.868)∗∗∗	1.041 (1.012: 1.070)∗∗
Internal medicine	0.842 (0.809: 0.876)∗∗∗	1.150 (1.087: 1.216)∗∗∗
Otorhinolaryngology	0.676 (0.630: 0.727)∗∗∗	1.202 (1.127: 1.283)∗∗∗
Pediatrics	0.573 (0.520: 0.632)∗∗∗	1.463 (1.359: 1.575)∗∗∗
Neuro psychiatry	0.915 (0.889: 0.942)∗∗∗	1.008 (0.996: 1.020)
Orthopedic surgery	0.901 (0.891: 0.911)∗∗∗	1.001 (0.991: 1.010)
Inpatient		
Internal medicine	0.886 (0.868: 0.905)∗∗∗	1.074 (1.046: 1.103)∗∗∗
Otorhinolaryngology	0.856 (0.838: 0.874)∗∗∗	0.939 (0.905: 0.974)∗∗
Pediatrics	0.609 (0.548: 0.677)∗∗∗	1.103 (1.039: 1.171)∗∗
Obstetrics gynecology	0.987 (0.966: 1.008)	0.598 (0.573: 0.624)∗∗∗
Neuro psychiatry	0.842 (0.823: 0.861)∗∗∗	1.236 (1.207: 1.266)∗∗∗
Orthopedic surgery	0.945 (0.933: 0.958)∗∗∗	0.988 (0.977: 1.000)∗∗
General surgery	0.981 (0.969: 0.995)∗∗	0.747 (0.730: 0.765)∗∗∗

∗p < 0.1, ∗∗p < 0.05, ∗∗∗p < 0.001.

**Table 9:** Adjusted changes in healthcare services utilization due to COVID-19 and achieving vaccination rate of 70%, stratified by type of health facilities.

	Onset of COVID-19 January 2020	Recovery period October 2021 (Achieving second dose vaccination rate 70%)
	IRR (95% CI)	IRR (95% CI)
Outpatient		
Level 3 Hospitals	0.876 (0.865: 0.886)∗∗∗	1.004 (0.995: 1.013)
Level 2 Hospitals	0.844 (0.816: 0.872)∗∗∗	1.298 (1.241: 1.357)∗∗∗
Level 1 Hospitals	0.801 (0.773: 0.829)∗∗∗	1.240 (1.179: 1.304)∗∗∗
Clinics	0.773 (0.704: 0.849)∗∗∗	1.541 (1.370: 1.734)∗∗∗
Long-term care facilities	0.848 (0.825: 0.872)∗∗∗	1.071 (1.040: 1.103)∗∗∗
Public Health Centers	0.766 (0.735: 0.799)∗∗∗	0.984 (0.953: 1.016)
Pharmacies	0.844 (0.820: 0.868)∗∗∗	1.055 (1.026: 1.086)∗∗∗
Inpatient		
Level 3 Hospitals	0.920 (0.905: 0.936)∗∗∗	0.966 (0.953: 0.980)∗∗∗
Level 2 Hospitals	0.851 (0.830: 0.872)∗∗∗	1.045 (1.019: 1.072)∗∗
Level 1 Hospitals	0.877 (0.863: 0.891)∗∗∗	0.866 (0.849: 0.883)∗∗∗
Clinics	0.724 (0.671: 0.781)∗∗∗	1.125 (1.049: 1.206)∗∗
Long-term care facilities	0.999 (0.964: 1.034)	0.572 (0.555: 0.589)∗∗∗

∗p < 0.1, ∗∗p < 0.05, ∗∗∗p < 0.001.

**Table 10:** Adjusted changes in healthcare services utilization due to COVID-19 and achieving vaccination rate of 70%, stratified by avoidable and non-avoidable hospitalizations.

	Onset of COVID-19 January 2020	Recovery period October 2021 (Achieving second dose vaccination rate 70%)
	IRR (95% CI)	IRR (95% CI)
Inpatient		
Non-avoidable hospitalization	0.919 (0.907: 0.931)∗∗∗	0.889 (0.873: 0.905)∗∗∗
Avoidable hospitalization	0.758 (0.730: 0.788)∗∗∗	1.008 (0.981: 1.036)

∗p < 0.1, ∗∗p < 0.05, ∗∗∗p < 0.001.

Model findings reflect a statistically significant reduction of 15.7% (IRR: 84.3%, 95% CI: 81.9%–86.7%, p < 0.001) and 11.6% (IRR: 88.4%, 95% CI: 87.0%–89.9%, p < 0.001) for outpatient and inpatient services, respectively, comparing pre-pandemic and post-pandemic levels (Table 4). During the recovery phase after reaching second dose vaccination rate of 70% in the country, we observed a slight increase for outpatient services compared to January 2020, although a 9.1% reduction (IRR: 90.9%, 95% CI: 89.3%–92.5%; p < 0.001) for inpatient services was observed (Table 4). As shown in Fig. 1, both outpatient and inpatient utilizations quickly recovered after reaching the 70% vaccination coverage and rebounded to pre-pandemic levels as of December 2022. These effects were heterogenous across subtypes of sex, age group, income level, departments, health facilities, and avoidable and non-avoidable hospitalization as described below.

### Adjusted changes by sex

Female rates of visiting outpatient services deceased slightly more than rates for males in January 2020 compared to pre-pandemic levels (Table 5). However, after achieving 70% second dose vaccination coverage, we observed a slightly slower recovery of outpatient care use for males compared to females compared to the onset of COVID-19. For inpatient services, we observed a greater reduction for females compared to males after reaching 70% vaccination coverage. However, the recovery for females was faster than that of males for inpatient utilization.

### Adjusted changes by age group

Older adults (65 years old and above) had the least impact on both outpatient and inpatient utilization services for post-COVID-19. For ages 65–74, utilization decreased by just 5.7% for outpatient services with a 4.9% decrease for inpatient services (Table 6). Reflecting global trends, South Korea saw the greatest reduction in utilization for the youngest group (0–6 years old); a 45.2% reduction for outpatient visits and 35.9% for inpatient services, followed by age group 7–18, and age group 19–39. For ages 65–74, utilization decreased by a mere 5.7% for outpatient services with a 4.9% decrease for inpatient services (Table 6). During the recovery phase after the country had achieved 70% second dose vaccination rate, all age groups in general increased in outpatient healthcare utilization with the greatest increase exhibiting in the youngest two groups. All age groups rebounded to pre-pandemic levels with the fastest recovery seen for the over 65 years of age for inpatient services.

### Adjusted changes by income level

We also observed variations across insurance premium contribution levels which are partly reflective of income level. All insurance contribution groups reduced their outpatient and inpatient utilization services in January 2020 as shown in Table 7. However, Medicaid patients and lower contributing groups (<40th percentile) experienced the least reduction in outpatient utilization in January 2020. In October 2021, all groups increased their outpatient utilization ranging from 2.1% to 9.7% from January 2020. Inpatient services, however, experienced a reduction in January 2020 and continued to reduce in October 2021, although Medicaid patient groups were the only group that increased by 10%. The fastest increases during the recovery period were exhibited for higher income groups especially for inpatient services, although all income groups rebounded to the pre-pandemic levels (Fig. 1).

### Adjusted changes by department

The decreases of outpatient health services were the greatest in the pediatrics department and otorhinolaryngology (ENT) department in January 2020 compared to pre-pandemic levels (Table 8). However, most of the departments for outpatient services rebounded to pre-pandemic levels compared to January 2020 with statistically significant increases for pediatrics and ENT departments. Reductions of inpatient health service utilization were also significant, the top three being pediatrics, neuro psychiatry departments, and otorhinolaryngology (ENT) at the onset of COVID-19. The restoration to pre-COVID inpatient utilization rates after achieving 70% vaccinations coverage seemed to be slower for departments compared to the onset of COVID-19, especially for OBGYN and general surgery departments.

### Adjusted changes by health facility

A 23.4% (IRR: 76.6%, 95% CI: 73.5%–79.9%, p < 0.001) and a 22.7% (IRR: 77.3%, 95% CI: 70.4%–84.9%, p < 0.001) statistically significant reduction in outpatient visits at public health centers and clinics, respectively, were observed in January 2020 (Table 9). The impact of the pandemic on higher level hospitals was the smallest for both outpatient and inpatient services compared to pre-COVID-19 levels. Outpatient medical use in long-term care facilities also decreased by 15.2% compared to the onset of COVID-19. In October 2021, all levels of health facilities for outpatient services increased compared to January 2020, except for public health centers, with the greatest increase shown for clinics. However, most inpatient utilization decreased in October 2021 compared to the onset of COVID-19 with the greatest reduction in long-term care facilities. However, the fastest increases in outpatient utilization during the recovery period was shown for long-term care facilities and pharmacies as shown in Fig. 1.

### Adjusted changes by avoidable and non-avoidable hospitalizations

Inpatient services can be divided into avoidable and non-avoidable hospitalizations. A 24.2% (IRR: 75.8%, 95% CI: 73.0%–78.8%; p < 0.001) and a 8.1% (IRR: 91.9%, 95% CI: 90.7%–93.1%; p < 0.001) statistically significant reductions were observed post-COVID-19 for avoidable hospitalization and non-avoidable hospitalization utilizations, respectively (Table 10). In October 2021, however, a statistically significant reduction of 11.1% was shown for non-avoidable hospitalization compared to the onset of COVID-19, although a slight increase in avoidable hospitalization was observed in October 2021. The greatest increases, however, was observed for non-avoidable hospitalization after October 2021.

### Robustness checks through falsification test

Falsification tests examined whether the ITS results were sensitive to the choice of breakpoints using two approaches.^26^, ^27^, ^28^ The first falsification test involved conducting a search for “data-driven” structural breaks in the data using a test for an unknown breakpoint.^26^ The data-driven test for a structural break detected two highly significant breaks at January 2020 and October 2021. These dates correspond to the onset of COVID-19 and the month of achieving 70% vaccination coverage in the country, respectively. The second falsification test validated the first test by examining the variability in statistical significance across a set of alternative pre-specified break points. All of the alternative breakpoints showed statistically significant p-values. Both suggested breakpoints were statistically significant (p < 0.001) indicating that the basic conclusions were not sensitive to a particular selection of breakpoint in early 2020 or in late 2022. These robustness checks provided insights into the validity of the stipulated break points and the appropriateness of the ITS design. Sensitivity analysis was further conducted to determine the if there were large differences in IRRs among other timepoints. Supplementary material C shows the result of the falsification test and sensitivity analysis.

## Discussion

Our study investigated the impact of changes in healthcare utilization attributable to COVID-19 and the COVID-19 vaccination by stratification including age, health facility type, income level, types of services, and avoidable/non-avoidable hospitalization. Findings were derived from the most comprehensive source of healthcare data available in South Korea. We find significant reductions in both monthly volume outpatient (−15.7%) and inpatient health service utilization (−11.6%) due to COVID-19 in early 2020 with similar trends observed for both adjusted and unadjusted figures. Possibly linked to South Korea's effective management and control, effective administration of COVID-19 vaccines, and health system resiliency, even at the peak of the Omicron surge, the total healthcare utilization rebounded above pre-pandemic levels as of December 2022.

We observed wide heterogeneity in the magnitude of relative changes in utilization across different types of services, age, sex, income level, health facility types, and avoidable/non-avoidable hospitalizations. This includes large variations in the changes in service utilization by income levels, with the least reduction shown for Medicaid patients and lower income groups compared to higher income groups. However, the fastest increases occurred for higher income group after October 2021. The youngest age groups experienced the largest drop possibly due to parents’ fear of infection,^30^ while ages 65 and above experienced a minimal impact on utilization partly due to relatively uninterrupted treatment for chronic diseases and effective management to accommodate patients who need regular check-ups and treatment along with a strong COVID-19 containment policy in the healthcare settings.^31^ However, inpatient utilization in long-term care facilities sharply decreased during the recovery period which could be contributed by the closures of nursing homes in 2021–2022.^32^ We also observed slightly greater reduction in women for both outpatient and inpatient services likely due to higher levels of fear reported among women.^33^ The reduction of health facility visits was the greatest for public health centers and continuously decreased during the recovery period, which may be partially due to shifting resources towards COVID-19 patients and fear of infection in lower-level health facilities.^31^^,^^34^^,^^35^ Public health centers are mostly utilized by the lower socioeconomic status and this continuous reduction in PHC utilization can imply inequalities in healthcare utilization among the vulnerable population in Korea. Although clinics experienced significant decreases at the onset of COVID-19, statistically significant increases were shown in October 2021 as many clinics were repurposed to offer testing and vaccination during the height of COVID-19. The results further indicate significant decreases in all departments for health care services with greatest reduction shown for pediatrics. Although vaccination helped to increase medical use with the greatest increases shown for pediatrics, not all departments had not recovered to pre-pandemic levels by December 2022, especially for OBGYN inpatient service. Lastly, we observed significant reductions in avoidable hospitalizations, which did not reach pre-pandemic levels.

This reduction of healthcare utilization during the pandemic can be attributed to a myriad of factors, which could stem from changes in health seeking behavior due to fear of cross-infection at the point of care, strict social distancing and isolation policies, and suspension of schools.^36^, ^37^, ^38^ However, the pandemic impact on lower healthcare utilization in Korea was less than that of regional peers and the global average rebounding above to pre-pandemic levels. In 2020 and 2021, the South Korean government had taken proactive and strategic measures instead of a lockdown to contain the pandemic and maintained services by establishing effective patient flow, such as triage and targeted referral of COVID-19 and non-COVID-19 patients,^5^^,^^13^ vaccinating majority of the population in just a few months, and expanding benefit packages to cover for COVID-19 related services and introducing teleconsultations.^37^^,^^39^^,^^40^ Post-MERS legislative and regulatory reforms enhanced the public health preparedness and system^18^^,^^41^ which contributed to its limited impact on the reduction of medical use in the country.

Groups of high vulnerability such as the elderly and those with limited income had protection. Contrary to global trends, outpatient and inpatient health care utilization among the elderly population (65 and older) in South Korea experienced the least reduction compared to other age groups. Although there were reductions in long-term care for the elderly, the preservation of acute care for the elderly is notable. Other commentators have credited the country's effective management of medical systems to along with a strong COVID-19 containment policy in healthcare settings.^31^ Similarly, South Korea's lower income groups experienced lesser magnitude of reduction in utilization due to the pandemic. However, their out-of-pocket spending and the occurrence of catastrophic health expenditure could be significantly higher in the near-poor group compared to other income groups and would be worth conducting further research.^42^

Most health service utilization rebounded to pre-pandemic levels as of December 2022 and possible factors include the high vaccination rate, the lifting of almost all levels of social distancing measures, and deliberate efforts to maintain the continuity of health services in the country. It is not possible for this analysis to untangle relative impact of the multiple related factors working together to restore service utilization in late 2022.

Although further research is needed to better understand the full health effects of COVID-19-related disruptions in utilization, it is well known that avoidance of and delayed access to health services was associated with poor health outcomes and may cause the occurrence and worsening of diseases and complications.^30^^,^^43^, ^44^, ^45^ Several studies reported that reductions in the use of in preventative health care services such as cancer screening and chronic disease management have a considerable impact on cancer incidence, prognosis,^43^^,^^46^, ^47^, ^48^, ^49^ and higher risk for the severity and implications for chronic diseases. Innovative ways to deliver health services such as telemedicine coupled with innovative financing incentives, more generous benefit packages, and adaptation to public financial management and provider payment methods will allow for resilient health systems and help adapt to crisis situations.^50^

To our knowledge, this is the first study to examine how Korea's healthcare utilization evolved due to COVID-19 to this granular level. The study is comprehensive as it covers the entire population across 7 years, stratified by sub-group categories, and uses a robust statistical method. It is important for policymakers to understand causes of utilization changes in population groups that were significantly impacted to make decisions on health policies for better and fairer healthcare systems and to adequately prepare and respond to a future pandemic. Our study provides new insight into South Korea's ‘relative’ preservation of healthcare utilization and may help policymakers and researchers in other countries to consider strategies that could curtail about the impact of COVID-19 and that of future pandemics on healthcare utilization.

The results are subject to three important limitations. First, the data is aggregated which may potentially mask important nuances that may be present in the underlying individual level data. Second, the data did not disaggregate between COVID-19 services and non-COVID-19 services, thus we were not able to explore the direct contribution of COVID-19 caseloads to care-seeking. Third, there is a possibility of data collection errors and delay in reporting, which may have impacted the results. For instance, NHIS reported that November to December 2022 medical utilization data may be subject to errors due to incomplete validation from the verification department. Fourth, the health insurance claims data will not include utilizations that are not covered by health insurance.

In conclusion, our study showed that COVID-19 significantly impacted healthcare utilization with a strong rebound. The study also showed heterogeneity in the impact among certain sub-groups population, but a remarkable ability to protect the access to care of vulnerable groups like the elderly and those with lower income. Preserving the mechanisms behind the institutional capacity of the country's preparedness and response to the pandemic should be a key interest for South Korean policymakers. Those in the global community eager to learn strategies and policies to shield vulnerable groups during future crises would also benefit from more detailed attention to the South Korean record to prepare for potential future pandemics.

## Contributors

Katelyn J. Yoo conceptualized and wrote the first draft, conducted the analysis, analyzed and interpreted data, and edited and reviewed drafted materials.

YoonKyoung Lee assisted literature review, conducted data analysis and interpretation, and reviewed drafted materials.

Seulbi Lee assisted with data collection and formatting, reviewed all citations and reviewed drafted materials.

Rocco Friebel contributed to shaping the storyline, edited and provided feedback on data interpretation and all drafts.

Soon-ae Shin led the collection of the data and reviewed drafted materials.

David Bishai contributed to study design, provided feedback on data interpretation and edited and provided feedback on all drafts.

Taejin Lee contributed to study design, provided feedback on data interpretation and provided feedback all drafts.

## Data sharing statement

Healthcare utilization data is deemed “sensitive” data in South Korea by the Personal Information Protection Act (PIPA). The data cannot be publicly shared because of data privacy restriction which protects patient confidentiality. Access to data is possible only once approval from the South Korean government and National Health Insurance Service (NHIS) has been obtained.

## Editor note

The Lancet Group takes a neutral position with respect to territorial claims in published maps and institutional affiliations.

## Declaration of interests

The authors whose names are listed immediately above certify that they have NO affiliations with or involvement in any organization or entity with any financial interest (such as honoraria; educational grants; participation in speakers’ bureaus; membership, employment, consultancies, stock ownership, or other equity interest; and expert testimony or patent-licensing arrangements), or non-financial interest (such as personal or professional relationships, affiliations, knowledge or beliefs) in the subject matter or materials discussed in this manuscript.

## Appendix A Supplementary data

Supplementary Final

## Footnotes

h Avoidable Hospitalization Trends From Ambulatory Care-Sensitive Conditions in the Public Health System in Mexico; Page A, Ambrose S, Glover J, Hetzel D. Atlas of avoidable hospitalizations in Australia: Ambulatory Care-Sensitive Conditions. Adelaide, SA: PHIDU, University of Adelaide (2007); Predicting Potentially Avoidable Hospitalizations (Gao, Jian PhD; et al, 2014).
