# Supplementary material for: The road to recovery: impact of COVID-19 on healthcare utilization in South Korea in 2016–2022 using an interrupted time-series analysis
Source: Lancet Reg Health West Pac. 2023 Sep 21;41:100904. doi: 10.1016/j.lanwpc.2023.100904 (PMC10541464; doi:10.1016/j.lanwpc.2023.100904)
Supplement: Supplementary Final [file mmc1.docx]

**Supplementary Materials**

**Supplement A Potentially avoidable hospitalizations**

| **Category** | **ICD10 codes (Purdy)** | **ICD 10 codes** |
| --- | --- | --- |
| **Vaccine preventable** | | |
| Influenza and pneumonia (I&P) | J10, J11, J13, J14, J15.3, J15.4, J15.7, J15.9, J16.8, J18.1, J18, J189, J120, J121, J122, J128, J129, J160, A481, A70x | J13-J14, J15.3–J15.4, J15.8–J15.9, J18.1 |
| Other vaccine-preventable diseases (OVPD) | A35, A36, A37, A80, B05, B06, B16.1, B16.9, B18.0, B18.1, B26, G00.0, M01.4 | A33–37, A95, B16, B05–B06, B26, G00.0, A17.0, A19 |
| **Chronic** |  |  |
| Diabetes complications (DC) | E10.0–E10.8, E11.0–E11.8, E12.0–E12.8, E13.0–E13.8, E14.0–E14.8, E139, E149 | E10–E14 |
| Nutritional deficiency (NUT) | E40, E41, E42, E43, E55.0, E64.3 | E40–E46, E50–E64 |
| Iron-deficiency anemia (AN) | D50.1, D50.8, D50.9, D460, D461, D463, D464, D510–D513, D518, D520, D521, D528, D529, D531, D571, D580, D581, D590–D592, D599, D601, D608, D609, D610, D611, D640–D644, D648 | D50 |
| Hypertension (HYPERT) | I10, I11.9 | I10–I11 |
| Congestive heart failure (HEART) | I11.0, I50, J81, I130, I255 | I50, J81 |
| Angina (ANG) | I20, I24.0, I24.8, I24.9, I25, R072, R073, R074, Z034, Z035 | I20 |
| Chronic obstructive pulmonary disease (COPD) | J20, J41, J42, J43, J47, J44, J40X |  |
| Asthma (ASTH) | J45, J46 | J45–J46 |
| **Acute** | | |
| Dehydration and gastroenteritis (GASTRO) | E86, K52.2, K52.8, K52.9, A020, A04, A059, A072, A080, A081, A083, A084, A085, A09, K520, K521 | E86, A00–A09 |
| Convulsions and epilepsy (EPILEP) | G40, G41, R560, O15, G253, R568 | G40–G41 |
| Ear, nose and throat infections (EN&T INFEC) | H66, H67, J02, J03, J040, J06, J31.2 | J20, J21, J40–J44, J47 |
| Dental conditions (DENTAL) | A69.0, K02, K03, K04, K05, K06, K08, K09.8, K09.9, K12, K13 |  |
| Perforated or bleeding ulcer (ULCER) | K25.0–K25.2, K25.4–K25.6, K26.0–K26.2, K26.4–K26.6, K27.0–K27.2, K27.4–K27.6, K28.0–K28.2, K28.4–K28.6, K920, K921, K922, K20x, K210, K219, K221, K226 | K25–K28, K92.0, K92.1, K92.2 |
| Pyelonephritis (PYELO) | N10, N11, N12, N13.6, N300, N390, N159, N308, N309 | N10–N12, N30, N34, N39.0 |
| Pelvic inflammatory disease (PELVIC) | N70, N73, N74 | N70–N73, N75–N76 |
| Cellulitis (CELL) | L03, L04, L08.0, L08.8, L08.9, L88, L98.0, I891, L010, L011, L020–L024, L028, L029 | A46, L01–L04, L08 |
| Gangrene (GAN) | R02 |  |
| Avoidable Conditions (AC) |  | A15–A16, A18, A17.1–A17.9, I00–I02,  A51–A53, B50–B54, B77 |
| Diseases related with the prenatal health care of  pregnancy and delivery (DPCPD) |  | O23, AS0, P35.0 |

Source: Avoidable Hospitalization Trends From Ambulatory Care-Sensitive Conditions in the Public Health System in Mexico; Page A, Ambrose S, Glover J, Hetzel D. Atlas of avoidable hospitalizations in Australia: Ambulatory Care-Sensitive Conditions. Adelaide, SA: PHIDU, University of Adelaide (2007); Predicting Potentially Avoidable Hospitalizations (Gao, Jian PhD; et al, 2014)

**Supplement B Epidemiology and Timeline of COVID-19 Control Policies**

**1. Jan 2020 – Dec 2020**


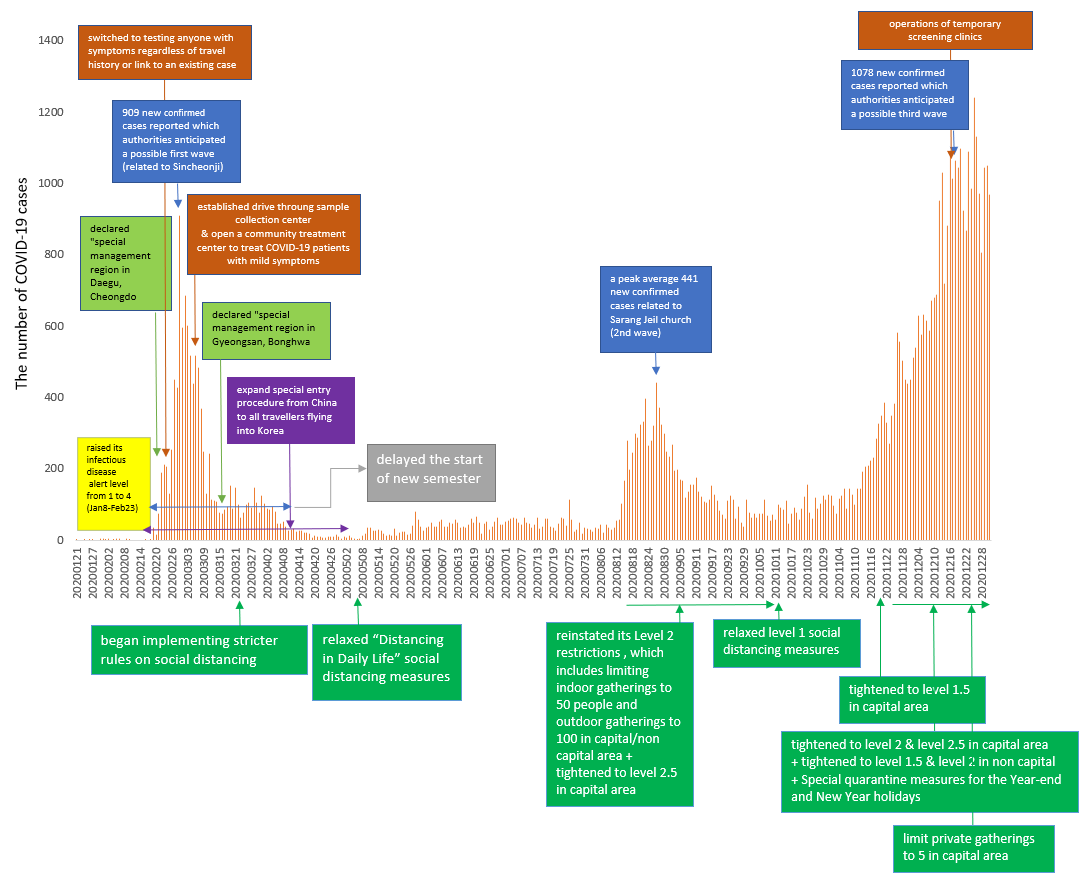


**2. Jan 2021 – Jan 2022 (before Omicron)**


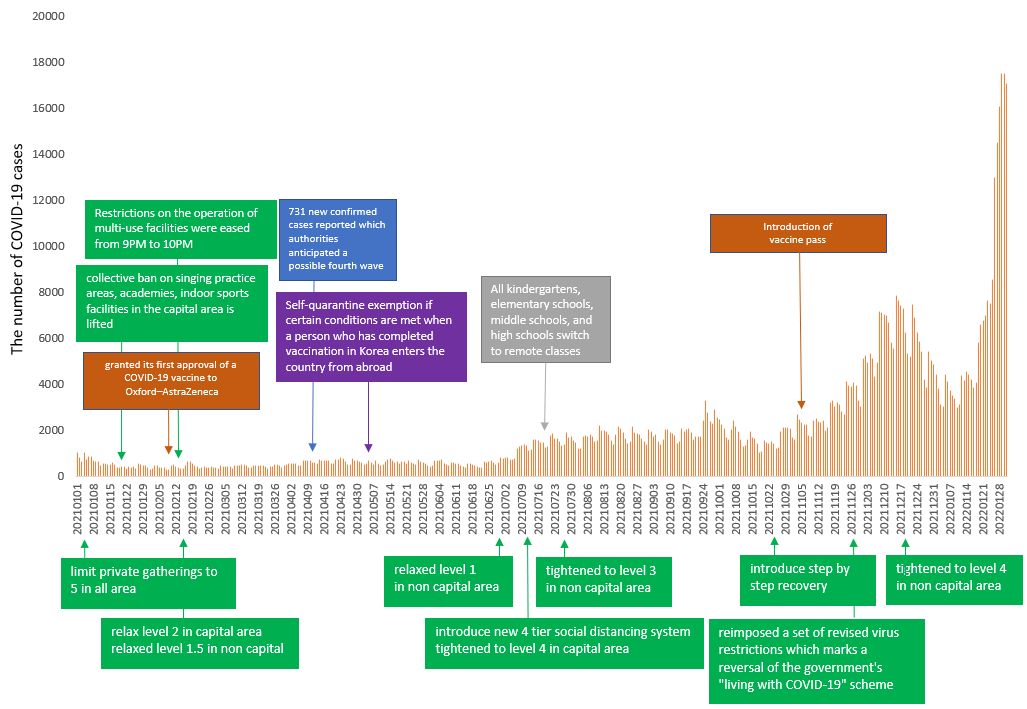


**3. Feb 2022 – May 2023 (after Omicron)**


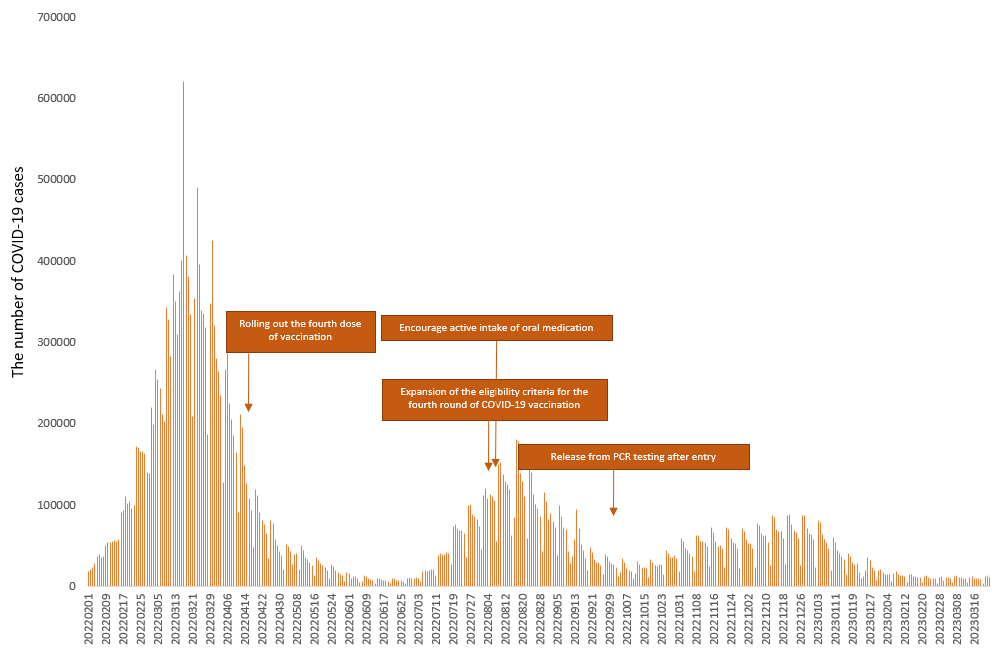


**Supplement C Falsification test results**

**1. Test for multiple breaks at *unknown* breaks**

Stata code : xtbreak test uo, breakconstant breaks(2) hypothesis(1)

|  | Bai & Perron Critical Values | | | |
| --- | --- | --- | --- | --- |
|  | Test  Statistic | 1% Critical  Value | 5% Critical  Value | 10% Critical  Value |
| SupW(tau) | 0.81 | 9.36 | 7.22 | 6.28 |

Estimated break points : 49 70

We conduct a falsification test for multiple breaks when we assume that we do not know the breakpoints. Our study coded January 2016 as time = 1. The results above indicate that the two statistically significant breakpoints are at time = 49 and 70 break points which corresponds to January 2020 and October 2021, respectively.

**2. Test for multiple breaks at *known* breaks**

Stata code : xtbreak test uo time, breakpoint(49 70, index)

H0 : no breaks vs. H1 : 2 breaks

|  | value |
| --- | --- |
| W(tau) | 48.67 |
| p-value (F) | 0.00 |

The second method tests for multiple breaks at known breaks, assuming the January 2020 and October 2021 were the given breakpoints as shown above. Both suggested breakpoints were statistically significant (p <0.001) rejecting the null hypothesis of no break point provides evidence that the intervention had no effect.

**3. Test for single break at *known* breaks**

1) Stata code : xtbreak test uo time, breakpoint(70, index)

H0 : no breaks vs. H1 : 1 break

|  | value |
| --- | --- |
| W(tau) | 38.28 |
| p-value (F) | 0.00 |

2) Stata code : xtbreak test uo time, breakpoint(49, index)

H0 : no breaks vs. H1 : 1 break

|  | value |
| --- | --- |
| W(tau) | 29.59 |
| p-value (F) | 0.00 |

Both breakpoints at January 2020 and October 2021 show statistically significant results.

**4. IRR by month (Health facility)**

|  | Tertiary | General | Hospital | Convalescent | Clinics | PHC | Pharmacy |
| --- | --- | --- | --- | --- | --- | --- | --- |
| '20.01 | 1.02 | 1.02 | 0.89 | 1.27 | 0.91 | 0.58 | 0.88 |
| '20.02 | 0.94 | 0.93 | 0.80 | 1.19 | 0.83 | 0.47 | 0.79 |
| '20.03 | 0.88 | 0.86 | 0.74 | 1.09 | 0.76 | 0.40 | 0.73 |
| '20.04 | 1.04 | 1.02 | 0.90 | 1.32 | 0.90 | 0.47 | 0.86 |
| '20.05 | 1.04 | 1.06 | 0.97 | 1.44 | 0.98 | 0.55 | 0.95 |
| '20.06 | 1.11 | 1.10 | 0.96 | 1.40 | 0.97 | 0.53 | 0.93 |
| '20.07 | 1.03 | 1.02 | 0.90 | 1.32 | 0.91 | 0.49 | 0.87 |
| '20.08 | 0.98 | 0.99 | 0.87 | 1.27 | 0.88 | 0.47 | 0.85 |
| '20.09 | 1.07 | 1.05 | 0.93 | 1.38 | 0.97 | 0.53 | 0.93 |
| '20.10 | 1.04 | 1.02 | 0.91 | 1.33 | 0.91 | 0.49 | 0.88 |
| '20.11 | 1.05 | 1.04 | 0.93 | 1.37 | 0.94 | 0.51 | 0.91 |
| '20.12 | 1.06 | 1.03 | 0.91 | 1.38 | 0.92 | 0.50 | 0.89 |
| '21.01 | 1.03 | 1.02 | 0.91 | 1.36 | 0.92 | 0.49 | 0.88 |
| '21.02 | 1.11 | 1.12 | 1.01 | 1.50 | 1.02 | 0.56 | 0.99 |
| '21.03 | 1.12 | 1.12 | 1.03 | 1.53 | 1.04 | 0.56 | 1.01 |
| '21.04 | 1.12 | 1.13 | 1.03 | 1.56 | 1.05 | 0.57 | 1.02 |
| '21.05 | 1.07 | 1.07 | 0.95 | 1.36 | 0.96 | 0.51 | 0.92 |
| '21.06 | 1.18 | 1.19 | 1.07 | 1.53 | 1.08 | 0.63 | 1.05 |
| '21.07 | 1.04 | 1.08 | 0.98 | 1.37 | 0.99 | 0.58 | 0.96 |
| '21.08 | 1.05 | 1.08 | 0.99 | 1.34 | 1.00 | 0.60 | 0.98 |
| '21.09 | 1.04 | 1.08 | 1.03 | 1.32 | 1.05 | 0.65 | 1.04 |
| '21.10 | 1.05 | 1.05 | 1.04 | 1.31 | 1.05 | 0.64 | 1.03 |
| '21.11 | 1.07 | 1.07 | 1.00 | 1.28 | 1.05 | 0.71 | 1.04 |
| '21.12 | 1.04 | 1.14 | 1.03 | 1.30 | 0.98 | 0.67 | 0.96 |
| '22.01 | 0.88 | 1.05 | 1.02 | 1.29 | 1.02 | 0.71 | 1.02 |
| '22.02 | 0.99 | 1.25 | 1.24 | 1.57 | 1.20 | 0.79 | 1.11 |
| '22.03 | 1.30 | 1.49 | 2.18 | 1.38 | 1.34 | 0.98 | 1.34 |
| '22.04 | 0.99 | 0.71 | 0.74 | 2.13 | 0.96 | 0.78 | 0.97 |
| '22.05 | 1.01 | 0.89 | 0.83 | 0.70 | 0.91 | 0.86 | 0.92 |
| '22.06 | 1.01 | 0.97 | 0.94 | 0.98 | 0.97 | 0.79 | 0.97 |

**Supplement D Adjusted slope changes in healthcare services utilization due to COVID-19**
 **and achieving vaccination rate of 70%**

Table SE-1 Adjusted slope changes in healthcare services utilization due to COVID-19 and achieving vaccination rate of 70%

|  | Onset of COVID-19  January 2020 | Recovery Period  October 2021  (Achieving vaccination rate 70%) |
| --- | --- | --- |
|  | IRR (95% CI) | IRR (95% CI) |
| Outpatient | 0.999(0.997:1.001) | 1.007(1.004:1.010)*** |
| Inpatient | 1.003(1.002:1.005)*** | 1.007(1.004:1.009)*** |

*p<0.1, **p<0.05, ***p<0.001

Table SE-2 Adjusted slope changes in healthcare services utilization due to COVID-19 and achieving vaccination rate of 70%, stratified by sex

|  | Onset of COVID-19  January 2020 | Recovery Period  October 2021  (Achieving second dose vaccination rate 70%) | |
| --- | --- | --- | --- |
|  | IRR (95% CI) | | IRR (95% CI) |
| Outpatient |  | |  |
| Male | 0.999(0.997:1.001) | | 1.008(1.005:1.011)*** |
| Female | 1.000(0.998:1.002) | | 1.006(1.003:1.009)*** |
| Inpatient |  | |  |
| Male | 1.002(1.001:1.004)** | | 1.005(1.003:1.007)*** |
| Female | 1.004(1.002:1.005)*** | | 1.008(1.006:1.011)*** |

*p<0.1, **p<0.05, ***p<0.001

Table SE-3. Adjusted slope changes in healthcare services utilization due to COVID-19 and achieving vaccination rate of 70%, stratified by age

|  | Onset of COVID-19  January 2020 | Recovery Period  October 2021  (Achieving second dose vaccination rate 70%) | |
| --- | --- | --- | --- |
|  | IRR (95% CI) | | IRR (95% CI) |
| Outpatient |  | |  |
| 0-6 years | 0.995(0.989:1.000)* | | 1.034(1.028:1.040)*** |
| 7-18 years | 1.002(0.997:1.006) | | 1.020(1.015:1.026)*** |
| 19-39 years | 0.999(0.996:1.001) | | 1.007(1.003:1.011)** |
| 40-64 years | 0.999(0.998:1.001) | | 1.003(1.000-1.006)** |
| 65-74 years | 1.001(1.000:1.002)** | | 1.002(1.000:1.003)* |
| Over 75 years | 0.996(0.996:0.997)*** | | 1.004(1.003:1.005)*** |
| Inpatient |  | |  |
| 0-6 years | 1.003(0.998:1.008) | | 1.017(1.011:1.023)*** |
| 7-18 years | 1.009(1.004:1.014)** | | 1.006(1.000:1.012)* |
| 19-39 years | 1.005(1.001:1.009)** | | 1.000(0.996:1.004) |
| 40-64 years | 1.002(1.001:1.003)*** | | 1.002(1.001:1.004)** |
| 65-74 years | 1.002(1.001:1.003)*** | | 1.010(1.008:1.011)*** |
| Over 75 years | 0.999(0.998:1.000) | | 1.013(1.011:1.014)*** |

*p<0.1, **p<0.05, ***p<0.001

Table SE-4 Adjusted slope changes in healthcare services utilization due to COVID-19 and achieving vaccination rate of 70%, stratified by income levels

|  | | Onset of COVID-19  January 2020 | Recovery Period  October 2021  (Achieving second dose vaccination rate 70%) | | |
| --- | --- | --- | --- | --- | --- |
|  | | IRR (95% CI) | | IRR (95% CI) |  |
| Outpatient | |  | |  |  |
| Medicaid | | 1.000(1.000-1.002) | | 1.001(1.000-1.003)* |  |
| Self-  Insured | 0-20TH | 0.987(0.982:0.991)*** | | 1.011(1.009:1.013)*** |  |
|  | 20-40th | 1.017(1.011:1.022)*** | | 0.995(0.992:0.999)** |  |
|  | 40-60th | 1.000(0.998:1.002) | | 1.006(1.003:1.008) |  |
|  | 60-80th | 1.003(1.001:1.005)*** | | 1.005(1.002:1.007)** |  |
|  | 80-100th | 1.001(0.999:1.003) | | 1.006(1.003:1.009)*** |  |
| Employer-  insured | 0-20TH | 0.996(0.994:0.998)*** | | 1.008(1.005:1.011)*** |  |
|  | 20-40th | 1.003(1.000-1.005)* | | 1.004(1.001:1.007)** |  |
|  | 40-60th | 1.000(0.997:1.002) | | 1.007(1.0040:1.010)*** |  |
|  | 60-80th | 0.994(0.991:0.998)** | | 1.017(1.013:1.021)*** |  |
|  | 80-100th | 0.998(0.996:1.000) | | 1.009(1.006:1.012)*** |  |
| Inpatient | |  | |  |  |
| Medicaid | | 1.005(1.004:1.006)*** | | 0.996(0.995:0.998)*** |  |
| Self-  insured | 0-20^TH^ | 0.990(0.986:0.994)*** | | 1.012(1.011:1.014)*** |  |
|  | 20-40^th^ | 1.020(1.015:1.026)*** | | 0.993(0.991:0.995)*** |  |
|  | 40-60^th^ | 1.003(1.002:1.005)*** | | 1.007(1.004:1.009)*** |  |
|  | 60-80^th^ | 1.006(1.005:1.008)*** | | 1.005(1.003:1.007)*** |  |
|  | 80-100^th^ | 1.004(1.003:1.005)*** | | 1.065(1.053:1.077)*** |  |
| Employer-  insured | 0-20^TH^ | 1.000(0.999:1.001) | | 1.009(1.006:1.011)*** |  |
|  | 20-40^th^ | 1.007(1.004:1.009)*** | | 1.003(1.001:1.006)** |  |
|  | 40-60^th^ | 1.004(1.002-1.006)*** | | 1.007(1.004-1.009)*** |  |
|  | 60-80^th^ | 0.999(0.996:1.002) | | 1.011(1.007:1.016)*** |  |
|  | 80-100^th^ | 1.001(0.999:1.003)* | | 1.010(1.007:1.012)*** |  |

*p<0.1, **p<0.05, ***p<0.001

Table 3E-5 Adjusted slope changes in healthcare services utilization due to COVID-19 and achieving vaccination rate of 70%, stratified by department (services)

|  | Onset of COVID-19  January 2020 | Recovery Period  October 2021  (Achieving second dose vaccination rate 70%) | |
| --- | --- | --- | --- |
|  | IRR (95% CI) | | IRR (95% CI) |
| Outpatient |  | |  |
| General | 0.997(0.995:1.000)** | | 1.014(1.011:1.017)*** |
| Internal medicine | 1.001(0.998:1.003) | | 1.006(1.001:1.012)** |
| Otorhinolaryngology | 0.988(0.983:0.993)*** | | 1.038(1.031:1.044)*** |
| Pediatrics | 0.999(0.994:1.005) | | 1.015(1.001:1.022)*** |
| Neuro Psychiatry | 0.997(0.996:0.999)*** | | 0.995(0.994:0.996)*** |
| Orthopedic Surgery | 1.002(1.002:1.003)*** | | 0.996(0.995:0.997)*** |
| Inpatient |  | |  |
| Internal Medicine | 1.006(1.004:1.008)*** | | 1.006(1.004:1.008)*** |
| Otorhinolaryngology | 0.995(0.993:0.997)*** | | 1.012(1.008:1.015)*** |
| Pediatrics | 1.002(0.996:1.008) | | 1.025(1.018:1.031)*** |
| Obstetrics Gynecology | 1.003(1.001:1.004)*** | | 1.036(1.031:1.040)*** |
| Neuro Psychiatry | 1.007(1.005:1.008)*** | | 0.988(0.985:0.990)*** |
| Orthopedic Surgery | 0.998(0.997:0.999)** | | 1.003(1.002:1.004)*** |
| Surgery | 0.999(0.998:1.000) | | 1.019(1.016:1.021)*** |

*p<0.1, **p<0.05, ***p<0.001

Table SE-6 Adjusted slope changes in healthcare services utilization due to COVID-19 and achieving vaccination rate of 70%, stratified by type of health facilities

|  | Onset of COVID-19  January 2020 | Recovery Period  October 2021  (Achieving second dose vaccination rate 70%) | |
| --- | --- | --- | --- |
|  | IRR (95% CI) | | IRR (95% CI) |
| Outpatient |  | |  |
| Level 3 Hospitals | 1.010(1009-1.011)*** | | 0.986(0.986-0.987)*** |
| Level 2 Hospitals | 1.005(1.003-1.006)*** | | 0.975(0.971-0.979)*** |
| Level 1 Hospitals | 1.001(1.000-1.003)* | | 0.989(0.984:0.994)*** |
| Clinics | 1.012(1.007:1.017)*** | | 0.975(0.965:0.984)*** |
| Long-term care facilities | 0.999(0.997:1.001) | | 1.010(1.007:1.012)*** |
| Public Health Centers | 0.975(0.971:0.978)*** | | 1.048(1.043:1.053)*** |
| Pharmacies | 0.998(0.995:1.000)** | | 1.014(1.011:1.017)*** |
| Inpatient |  | |  |
| Level 3 Hospitals | 0.998(0.995:1.000)** | | 1.014(1.011:1.017)*** |
| Level 2 Hospitals | 1.004(1.003:1.006)*** | | 0.997(0.996:0.999)*** |
| Level 1 Hospitals | 0.997(0.996:0.999)*** | | 1.024(1.021:1.026)*** |
| Clinics | 1.010(1.005:1.016)*** | | 1.050(1.043:1.057)*** |
| Long-term care facilities | 0.998(0.995:1.001) | | 0.984(0.981:0.987)*** |

*p<0.1, **p<0.05, ***p<0.001

Table SE-7 Adjusted slope changes in healthcare services utilization due to COVID-19 and achieving vaccination rate of 70%, stratified by avoidable and non-avoidable hospitalizations

|  | Onset of COVID-19  January 2020 | Recovery Period  October 2021  (Achieving second dose vaccination rate 70%) | |
| --- | --- | --- | --- |
|  | IRR (95% CI) | | IRR (95% CI) |
| Inpatient |  | |  |
| Non-avoidable hospitalization | **1.003(1.003:1.005)***** | | **1.005(1.003:1.007)***** |
| Avoidable hospitalization | **1.000(0.997:1.002)** | | **1.015(1.011:1.018)***** |

*p<0.1, **p<0.05, ***p<0.001
